# Role and Function of T Cell-Derived Exosomes and Their Therapeutic Value

**DOI:** 10.1155/2021/8481013

**Published:** 2021-11-12

**Authors:** Yuanyuan Shao, Xiaofeng Pan, Rong Fu

**Affiliations:** Department of Hematopathology, Tianjin Medical University General Hospital, 154 Anshan Street, Tianjin 300052, China

## Abstract

Exosomes are membrane-bound extracellular vesicles that are produced in the endosomal compartment of most eukaryotic cells. Containing proteins, RNA, and DNA, exosomes mediate intercellular communication between different cell types by transferring their contents and thus are involved in numerous physiological and pathological processes. T cells are an indispensable part of adaptive immunity, and the functions of T cell-derived exosomes have been widely studied. In the more than three decades since the discovery of exosomes, several studies have revealed that T cell-derived exosomes play a novel role in cell-to-cell signaling, especially in inflammatory responses, autoimmunity, and infectious diseases. In this review, we will summarize the function of T cell-derived exosomes and their therapeutic potential.

## 1. Introduction

In 1982, Harding et al. discovered exosomes while observing the maturation of mammalian reticulocytes [1]; subsequently, Johnstone et al. coined the term “exosome” in 1987 [2, 3]. They observed the accumulation of “membrane-bound vesicles” in the lumen of multivesicular endosomes (MVE) as a result of the inward invagination of its limiting membrane. After the infusion of MVE with the cellular membrane, “vesicular inclusions” were released into the extracellular space. As a comparison with exosomes, ectosomes are released from the plasma membrane as shedding vesicles.

Exosomes are membrane-bounded extracellular vesicles (EVs) usually of 30 nm to 150 nm in diameter produced in the endosomal compartment of most eukaryotic cells [4]. According to Exocarta (http://www.exocarta.org), an online database of exosomes, more than 9760 proteins (from the cytoplasm, membrane, Golgi apparatus, and reticulum), as well as more than 3000 mRNAs and have identified as exosomal cargo [5, 6]. Suppressive or promotive factors can be transferred by T cell-derived exosomes to other cells *via* membrane vesicle trafficking, thus influencing a variety of immune responses, including adaptive immunity and immune responses to pathogens and cancer [7, 8].

### 1.1. The Function of T Cell-Derived Exosomes

The process of exosome secretion in T lymphocytes can be initiated by increased intracellular Ca^2+^ concentration, which is induced by external stimuli [9]. The exosomes released by activated human T lymphocytes are characterized by bioactive Fas ligand (FasL) and APO ligand in their aqueous core, which promote activation-induced cell death [10]. Jurkat T cell-derived exosomes constitutively express natural-killer group 2 member D (NKG2D) ligand, which inhibits natural killer cell (NK) cytotoxicity during thermal and oxidative stress [11]. It has also been found that exosomes derived from Jurkat T cells can regulate proliferation and the physiological functions of endothelial cells, primarily through a CD47-dependent pathway [12]. Structural studies of T lymphocyte-derived exosomes have yielded significant insights into their typical surface molecules, which include glucocorticoid-induced tumor necrosis factor receptor (GITR) [13], major histocompatibility complex (MHC)-I and MHC-II [14], lymphocyte function-associated antigen- (LFA-) 1/2 [15], tumor susceptibility gene 101 (TSG101) [16], APO-1/CD95 (Fas), FasL [17], C-X-C motif chemokine receptor 4 (CXCR4) [18], and T cell receptor (TCR) [19].

Proteomics studies have shown that there are some constant proteins in most T cell-derived exosomes, including glyceraldehyde-3-phosphate dehydrogenase (GAPDH), enolase, specific heat-shock proteins, CD81, CD63, major luminal proteins (including tubulin isoforms and actin), annexins, and proteins involved in immunological processes such as human leukocyte antigen-I (HLA-I), components of the TCR/CD3 complex, *β*2-microglobulin, and specific integrins [20, 21]. Studies have also detected membrane-associated ATPase valosin-containing proteins (VCP) in leukemic T cells, but not normal T cells from healthy donors [20]. Moreover, some miRNAs with regulatory functions are also present in exosomes, with several specifically enriched miRNAs. The activity of the major exosomal heterogeneous nuclear riboprotein A2/B1 largely contributes to this phenomenon [22]. Recent studies have found that exosomal DNA can also be present in T cell-derived exosomes [23].

During T cell activation, a specific set of tRNA fragments (tRFs) derived from the 5′-terminus and 3′-internal region of tRNAs without variable loops are released in exosomes; 45% of tRFs but fewer than 1% of miRNAs from the corresponding cellular RNAs were significantly enriched in exosomes. And T cell activation was blocked when this process was inhibited [24].

CD3+ T cell-derived exosomes can stimulate and promote the proliferation of resting CD3+ T cells. These exosomes can also induce a relative increase in CD8+ T cells with the help of interleukin-2. Moreover, these exosomes may inhibit HIV through human chemokine (C-C Motif) ligand 5 (CCL5)/regulated upon activation normal T cell expressed and secreted (RANTES) [24].

The function of exosomes may resemble their cell of origin. T cells can secrete CD63+ exosomes to antigen-presenting cells (APCs) during immune synapse formation, which causes an antigen-driven unidirectional transfer of miRNAs. This process is highly dependent on sphingomyelinase-2 [8]. And this immunological synapse can promote the efficiency of exosome transfer, mostly in a neutral sphingomyelinase-2-dependent pathway.

Moreover, T cell-derived exosomes can prime dendritic cells (DCs) for antiviral responses. Antigen-bearing DCs initiate antipathogen programs when they form immunological synapses with T cells. The exosomes can induce antiviral responses through genomic and mitochondrial DNA. After fusion with target cells, they can activate the cyclic GMP-AMP synthase (cGAS)/signaling adaptor protein (STING) cytosolic DNA-sensing pathway and induce the expression of interferon regulatory factor 3- (IRF3-) dependent interferon regulated genes. Moreover, the DCs gain resistance to viral infection after exosome uptake [23]. Additionally, metastasis of esophageal cancer cells is promoted by T cell-derived exosomes from irradiated infiltrating esophageal carcinoma patients [25].

Exosomes derived from human T cells may participate in the pathogenesis of nonobese diabetes mellitus. These exosomes transfer the active form of microRNAs (miR-155, miR-142-5p, and miR-142-3p) to pancreatic *β* cells, inducing apoptosis by inhibiting the expression of genes involved in chemokine signaling (Ccl2, Ccl7, and Ccl10) exclusively in *β* cells. The recruited immune cells exacerbate *β* cell death when these genes are activated [26]. Exosomes may also show immunosuppressive properties that are against the function of the cell of origin. This phenomenon is presumably predicated by the type of target cell they interact with. For example, CD40L and inducible costimulator protein (ICOS) can be highly expressed after the activation of CD4+ T cells. Nevertheless, DCs will undergo apoptosis and even T cell responses will be silenced after binding of the main effector molecule FasL by DCs through CD54/LFA-1 and pMHV-II/TCR [27, 28].

### 1.2. T Cell-Derived Exosomes in Diseases

Using a novel method to isolate and characterize exosomes derived from T cells even in a limited amount of sample [29], exosomes have been found to be involved in the pathogenesis of numerous inflammatory diseases.

Oral lichen planus (OLP) is a chronic inflammatory disease with uncertain etiology. It is currently thought to be T cell mediated. In patients with OLP, T cell-derived exosomes that contain macrophage inflammatory protein- (MIP-) 1*α*/*β* can drive the trafficking of CD8+ T cells after binding with CC chemokine receptor (CCR)1/5, contributing to the development of OLP [30]. Moreover, T cell-derived exosomes from OLP patients exhibited an aberrant cytokine profile. An *in vitro* study showed that those aberrant cytokines can trigger apoptosis in keratinocytes [31].

Sjören's syndrome is a chronic autoimmune disease in which the body's leukocytes destroy moisture producing glands. In Sjören's syndrome patients, activated T cell-derived exosomes containing miR-142-3p can be transferred into glandular cells. miR-142-3p can alter intracellular Ca^2+^ signaling and destroys the protein production in salivary gland cells, thus directly impairing epithelial cell function [32]. Studies have also found that T cell-derived exosomes participate in the pathogenesis of hypertension (HTN). In an angiotensin-induced HTN animal model, CD3+ T cell-derived exosomes entered peripheral circulation and the kidneys. They also found that the concentration of exosomes was positively correlated with high blood pressure [33].

### 1.3. CD4+ T Cell-Derived Exosomes

CD4+ T cells can produce cytokines and interact with other cells such as NK cells, macrophages, and CD8+ T cells. The peptides presented by class II MHC can stimulate the TCR of CD4+ T cells to make them exert their functions, which include activating other immune cells. Th1 cells, which are CD4+ T cells against intracellular pathogens, can produce immunoglobulin G2A (IgG2a) antibodies to optimize the clearance of viruses and extracellular bacteria by stimulating the conversion of immunoglobulins in B cells.

Exosome secretion is promoted *in vitro* by CD28 and TCR costimulation. However, when other stimuli such as phorbol 12-myristate 13-acetate (PMA) and ionomycin were used, this effect was not found. This might be because cells produce exosomes as a physiological response [34]. Moreover, T cells can regulate the release of distinct exosome subpopulations under different activation statuses [35].

In ischemic heart disease patients, circulating exosomes containing miR-142-3p from activated CD4+ T cells can boost post ischemic ventricular remodeling by activating myofibroblasts, primarily *via* the phosphatidylserine receptor [36]. Activated T lymphocytes can also infiltrate atherosclerotic plaques in patients with ischemic heart disease. These exosomes can enhance cholesterol accumulation in monocytes, which is observed in cell coculture. Endogenous phosphatidylserine receptor facilitates exosome internalization. And the subsequent increase in the production of the proinflammatory cytokine TNF-*α* parallels cholesterol accumulation in monocytes [37].

Furthermore, specific proteins involved in the RAS signaling pathway are enriched in exosomes and can drive ERK phosphorylation in recipient immune cells [38]. Antigen-specific T cell exosomes may serve as a new type of immunosuppressive reagent that be used for transplantation rejection and treating autoimmune diseases [39].

CD4+ T cell-derived exosomes can also be applied in clinical practice as novel biomarkers for disease development. In chronic hepatitis B patients, exosomes derived from CD4+ and CD8+ T cells are released into serum, while in patients with nonalcoholic steatohepatitis (NASH) or nonalcoholic liver disease (NAFLD), high level of macrophage/monocyte-derived and invariant natural killer (iNKT) cell-derived exosomes are released into the serum [40].

Follicular helper T cells (Tfhs) constitute another subpopulation of CD4+ T cells that regulate the development of antigen-specific B cell immunity in the germinal center of secondary lymphoid follicles. Tfhs are closely involved in the development of antibody-mediated rejection (AMR) after renal transplantation. Tfhs-derived CD4+ CXCR5+ CXCR3- exosomes, which mediate B cell proliferation and differentiation, were significantly increased in the AMR group compared with those in the non-AMR group [41].

### 1.4. CD8+ T Cell-Derived Exosomes

CD8+ T cells play an essential role in protecting against infectious agents by killing infected cells following recognition of microbial peptides presented by MHC class I molecules on the surface of target cells. Major efforts are underway to harness tumor-specific CD8+ T cells to treat cancer cells. After target cell recognition, cytotoxic granules are released into the immunological synapse formed between killer cells and their targets.

The granules secreted by cytotoxic T lymphocytes (CTLs) contain perforins (referred to as membrane-perturbing proteins), granulysin, and granzymes. The perforin pore gives granzymes access to the cytosol of target cells, where they induce cell death pathways.

Exosomes derived from CD8+ T cells also contain granzymes and perforin. These granzymes can be directly transferred when CTLs fuse with the membrane of target cells or through endocytosis [42]. Typically, during immune synapse formation with target cells, exosomes are released from CTLs to assist in killing target cells [43].

Generally, CTLs secrete extracellular vesicles following antigen stimulation. If treated with IL-12, CTLs can secrete exosomes with various diameters that are selectively enriched for certain exosomal proteins. The proteins inside exosomes may display altered catalytic and binding activities [44]. Additionally, CTL-derived exosomes can strengthen the effects of weak antigen stimulation on CTLs and activate bystander CTLs in the absence of antigens.

Macrophages are a type of leukocyte that engulf and digest cancer cells, cellular debris, microbes, foreign substances, and anything that is recognized as “foreign protein” in the process of phagocytosis. Treatment with trinitrophenol- (TNP-) specific exosomes, which carry miRNA-150 derived from CD8+ T cells, impairs functional interactions between innate and adaptive immune responses *in vivo*, as well as phagocytic activity *in vitro* [45].

Moreover, macrophages lose contact sensitivity when co-cultured with CD8+ T cell-derived exosomes. At the same time, the proliferation of effector T cells may be inhibited and regulatory T cell (Treg) induction may be promoted [46]. CD8+ T cells deliver miRNA-150 *via* exosomes to antigen-primed macrophages, which suppresses delayed-type hypersensitivity in a mouse model [47]. Except for delivering cytotoxic molecules to targets, in patients with melanoma, the ERK and NF-*κ*B pathways are activated by CD8+ T cell-derived exosomes, resulting in the expression of matrix metalloproteinase (MMP) 9 and tumor cells becoming invasive *in vitro*. However, this does not affect tumor cell apoptosis or proliferation [48]. CD8+ T cell-derived exosomes with membrane expression of FasL can promote the invasion and metastasis of Fas+ tumor cells through MMP-9-mediated extracellular matrix degradation [45].

Exosomes from CD8+ T cells not only inhibit the CD8+ CTL responses mediated by antigen-specific DCs by affecting the function of target cells through the endocytosis of APCs and B cells but also inhibit antitumor immunity in an antigen-dependent manner [49]. Activated CD8+ T cells can secrete exosomes that interrupt fibroblastic stroma-mediated tumor cell invasion and metastasis by the apoptotic depletion of mesenchymal tumor stromal cells [47]. However, it has been argued that exosomes from exhausted CD8+ T cells possibly participate in the prevention of tumor growth, invasion, and metastasis [50]. Functional analysis has indicated that differently expressed lncRNAs from a variety of exosomal sources actively participate in regulating the diverse CD8+ T cell responses by altering biosynthetic processes, gene expression, and metabolism [51].

### 1.5. Treg-Derived Exosomes

Tregs are another type of CD4+ T cells with a prominent suppressive function rather than providing helper activity. These cells express CD3, CD4, and CD25 on their surface and are indispensable for specific immune tolerance [52, 53]. According to their origin, Tregs can be subdivided into “induced” regulatory T cells (iTregs) or thymically derived Tregs (tTregs). In the microenvironment, a variety of immune cells such as macrophages, DCs, NK T cells, CD4+ T cells, CD8+ T cells, and B cells are inhibited by various subtypes of Tregs. Tregs can specifically inhibit immune responses through several mechanisms. For example, Tregs can secrete suppressor cytokines including IL-10 and TGF-*β* [54, 55], consume the local IL-2 concentration, and induce apoptosis or cell cycle arrest through direct cellular contact.

Compared with other T cell subtypes, Tregs secrete more exosomes, with more membranous molecules such as CD25, CTLA4, and CD73. The Rab family proteins are indispensable for exosome release by T cells. It has been reported that Rab27b double-knockout (Rab27-DKO) Tregs cannot secrete exosomes, which makes them unable to suppress Th1 function [56]. Treg-derived exosomes primarily achieve their suppressive functions through CD73 and the subsequent release of adenosine to suppress inflammation [57–59].

Other functional molecules isolated from Treg-derived exosomes include let-7d, let-7b, and miR-155. These can be transferred to Th1 cells to inhibit their proliferation and cytokine release [56]. Thus, Treg-derived exosomes and their cargo may represent promising antitumor targets. Treg-derived exosomes can also inhibit DCs. When exosomes containing miR-142-3p and miR-150-5p are engulfed by DCs, the DCs will secrete less IL-10 and IL-6 following lipopolysaccharide (LPS) stimulation [60]. Treg-derived exosomes also regulate intracellular immunity, which can be applied to increase allograft kidney survival [61].

Tregs also inhibit cytotoxic T lymphocytes *via* exosomes, which can be reversed by GW4869, an EXO inhibitor. This inhibition is related to regulation of interferon and perforin [50]. Apart from tumor suppression, Treg-derived exosomes can prolong the survival of liver transplantation patients [61].

### 1.6. Chimeric Antigen Receptor T Cell-Derived Exosomes

On the basis of the basic characteristics of antigen recognition and immune cell signaling, chimeric antigen receptors (CARs) have been successfully designed and applied to treat malignant blood diseases.

CAR-T cell therapy comes with unprecedented efficacy in hematologic malignancies. However, after interacting with tumor cells, CAR-T cell-induced cytokine release syndrome (CRS) commonly manifests with high fever, nausea, tachycardia, hypotension, rash, headache, shortness of breath, and bone marrow suppression [62]. Patients may develop hypogammaglobulinemia and B cell aplasia as a result of the elimination of healthy B cells by CAR-T cells. However, these mild clinical manifestations are acceptable toxicities. When antibodies are injected or CAR-T cell therapy is combined with bone marrow transplantation, this clinical manifestation can be managed. However, CAR-T cell toxicity management is still a challenge, especially central nervous system toxicity.

**Figure 1:**
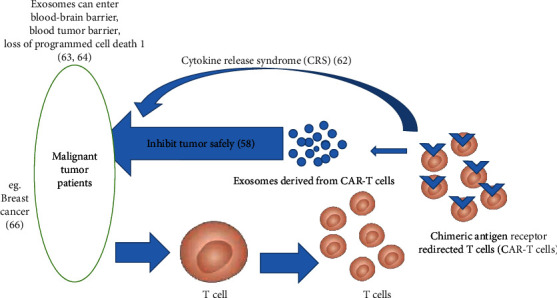
CAR-T cell-derived exosomes for tumor treatment.

Studies have found that exosomes derived from CAR-T cells display the functions of reducing the toxicity induced by CAR-T cells and can cross the blood-brain barrier and blood-tumor barrier [63], as well as loss of programmed cell death 1 (PD1) protein, which means that recombinant PD-L1 treatment fails to weaken the antitumor effect [64]. CAR-containing exosomes express high levels of cytotoxic molecules and inhibit tumor cells. This has been verified in a preclinical study, which showed its relative safety [58]. Anticancer drugs can kill target cells by being loaded into exosomes derived from CAR-T cells because of their superior potential compared with that of CAR-T cells to penetrate the extracellular matrix (ECM) of solid tumors [63, 65]. For instance, CAR-containing exosomes significantly inhibited the growth of both endogenous and exogenous mesothelin-positive triple-negative breast cancer cells without obvious side effects while displaying a high tumor inhibition rate (Figure 1) [66].

## 2. Conclusions

In conclusion, the current research into T cell-derived exosomes is still in the exploratory stage. Whether T cell-derived exosomes will have a major role in the future for clinical diagnosis or treatment remains to be determined. We reviewed CD4+ T cell-, CD8+ T cell-, and Treg-derived exosomes, as well as those from other issues. Better experimental models and systematic studies are needed, especially in autoimmune diseases, before T cell-derived exosomes can transition from the laboratory to the clinic.

## Data Availability

This review does not contain any original data. The data cited could be found in the reference papers.
